# Melanoma as a surprising solution to the puzzle of intestinal obstruction*

**DOI:** 10.1590/abd1806-4841.20164685

**Published:** 2016

**Authors:** Maksymilian Gajda, Grażyna Kamińska-Winciorek, Iwona Grzesiak, Jerzy Wydmański

**Affiliations:** 1 Clinical Oncology Ward, Starkiewicz Specialised Hospital – Dąbrowa Górnicza, Poland; 2 Maria Sklodowska-Curie Memorial Cancer Center and Institute of Oncology Gliwice - Branch Gliwice, Poland

**Keywords:** Colon, Gastrointestinal tract, Ileus, Intestinal obstruction, Laparotomy, Melanoma

## Abstract

We present a case of a 71-year-old man with an advanced melanoma of the right colon. The final diagnosis was determined based on histopathological examination of the material collected during urgent laparotomy performed due to ileus. Although we considered the tumor to be a disseminated primary melanoma of the colon, the possibility of unknown primary origin could not be excluded. Palliative chemotherapy and radiotherapy reduced symptoms associated with the disease and prolonged patient's survival.

## INTRODUCTION

Melanoma is a malignant neoplasm developing from melanocytes, mostly associated with the skin. Less frequently, such tumors can be found in other non-cutaneous locations such as the eye, middle ear, meninges and gastrointestinal, genitourinary and respiratory tracts.^1,2^ Review of over 84,800 cases of melanoma showed that 91.2% were cutaneous, 5.2% ocular, 2.2% of unknown primary site and only 1.3% in the gastrointestinal mucosa.^3^ In most cases, involvement of gastrointestinal tract (GIT) is metastatic, usually with 1% to 4% found in living patients and up to approximately 60% of the cases at autopsy.^4,5^ The primary skin lesion can be determined in most cases of GIT involvement.^6^ Primary colonic melanoma is a very rare and controversial clinical entity. Although optimal treatment guidelines for the disease have not been defined yet, we hope our experiences can contribute to the existing literature.

## CASE REPORT

**Figure 1:**
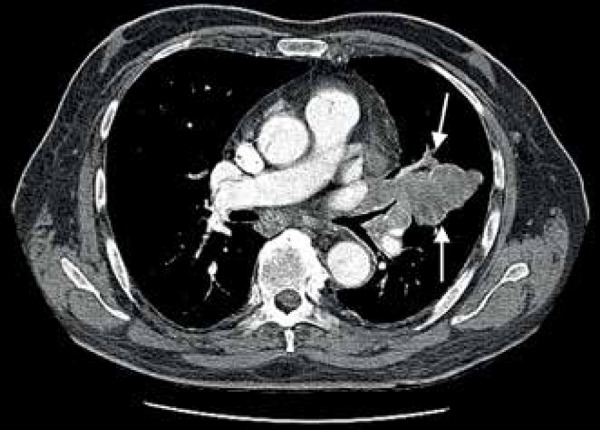
Postoperative thoracic CT. Metastatic tumor (arrows) located in the left lung

**Figure 2:**
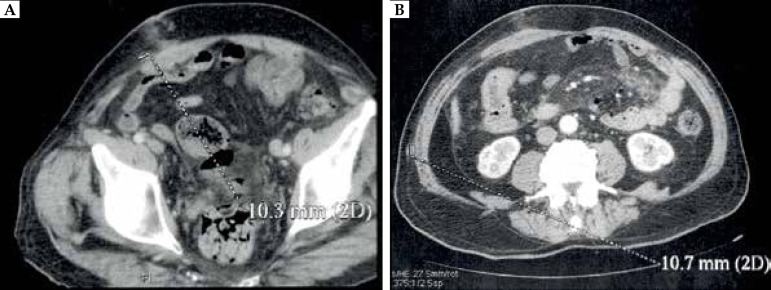
Postoperative abdominal CT. Subcutaneous metastases **a)** 10.3 mm; **b)** 10.7 mm in diameter

**Figure 3:**
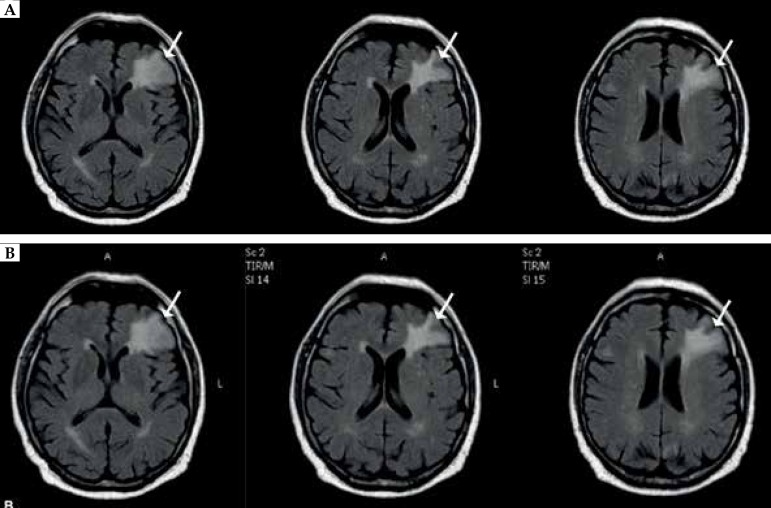
NMRI of the brain. Set of brain NMRI comparing the size of metastatic tumor (marked with arrows): **a)** before radiotherapy (upper row) **b)** after radiotherapy (lower row)

In 2011, a 71-year-old man presented to our clinic with diarrhea, 3-month history of vomiting and weight loss (6kg in 1.5 months). Fiberoptic colonoscopy examination revealed an extensive, exophytic neoplastic infiltration of the colon. Pathological examinations of the collected sample suggested an initial diagnosis of adenocarcinoma. Two weeks later, before the conclusion of the diagnostic process, the patient was submitted to an emergency right hemicolectomy due to ileus. Pathological examination revealed an ulcerated tumor infiltrating mucosa, submucosa and muscular layers with high expression of S-100 and HMB-45. Our final diagnosis was melanoma. Subsequent patient examination revealed no suspicious skin lesions. The patient also denied previous pigmented skin lesion. Postoperative thoracic computer tomography (CT) revealed metastatic tumors in the lungs (Figure 1). Abdominal CT also showed subcutaneous metastases (two of the lesions were approximately 10mm in diameter) (Figure 2). The patient was qualified for palliative systemic treatment with a regimen of dacarbazine (DTIC) after diagnosis of stage IV primary colonic melanoma (PCM). Physical examination performed on admission to first cycle of chemotherapy showed conglomerate of lymph nodes on the left axilla (4x3cm in size). After four cycles, chemotherapy was canceled because of metastasis demonstrated by nuclear magnetic resonance imaging (NMRI) of the brain and progression of the left axillary lymph nodes. The patient underwent whole-brain radiotherapy (30 Gy/10fx) (Figure 3A). Control brain NMRI showed stabilization (Figure 3B). After the operation, the patient reported no weakness (except during the last few weeks of life) or pain. He died 11 months after surgery.

## DISCUSSION

Melanoma develops from the malignant transformation of melanocytes or from cells capable of melanocytic differentiation. Although small and large intestines typically contain no melanocytes, a few theories try to explain the origin of melanoma in the bowel.^4,7^ The occurrence of primary colonic melanoma (PCM) – an extremely rare tumor of the gastrointestinal tract – could be explained by the fact that melanocytes have occasionally been found in the digestive and respiratory tracts.^7^ Tumor regression could be another explanation for the occurrence of "metastatic melanoma in the colon" with unknown primary site. Infection or other changes in the immune system can be associated with spontaneous regression of the melanomas' primary site.^4^ Identification of intracellular melanin is one of the key pathologic features of melanoma.^1^ When melanomas lack melanin pigmentation (amelanotic melanoma), diagnosis can be even more challenging. Immunohistochemical (IHC) analyses for S-100, HMB-45, Melan-A, microphthalmia transcription factor, tyrosinase, and Mart-1 are essential for the proper diagnosis of mucosal melanomas.^1^ Establishing differential diagnosis of melanoma is often a tough task and should include: metastatic melanoma; Paget's disease (cytokeratins); lymphoma (CD45 and CDs); undifferentiated carcinoma (chromogranin, synaptophysin); clear cell sarcoma; malignant peripheral nerve sheath tumor (MPNS – epithelioid variant showing S-100-positivity); gastrointestinal stromal tumor (CD117-positive); epithelioid leiomyosarcoma; and (as in our case) adenocarcinoma. Unlike melanoma of the skin, guidelines for PCM are lacking due to the limited number of reported cases. According to the limited data, surgical resection with wide margins appears to be the treatment of choice for PCM.^4,8^ When distant metastasis is present, surgery may have only a limited palliative role and be performed, as in the present case, on an emergency basis.^7,8^ Traditional chemotherapeutic agents, radiation therapy and, especially, new targeted therapy and immunotherapy have also been reported as palliative modalities for advanced melanomas.^1,4,8,9^ Both primary mucosal and metastatic melanomas are more aggressive than cutaneous melanomas and have poorer prognosis with median survivals of 4-6 months with an average 5-year survival rate of 20% or less.^1,7^ Bowel perforation and obstruction are associated with poor life expectancy (less than 10 months), as observed in our case.^4,7,10^ In cases with such alarming symptoms, melanoma might be a possible solution to the puzzle. Nevertheless, the outcome is usually poor, even for those identified in the early stages. Factors associated with decreased survival include advanced stage of the disease and hidden metastasis at diagnosis.^1,3^

To conclude, PCM is a very dynamic disease associated with poor treatment outcomes. In the present case, the combined palliative treatment reduced symptoms of the disease and prolonged patient survival. However, considering the apparent advanced stage of the primary melanoma of the colon, the possibility of a metastatic origin could not be excluded. The discussion as to whether such cases should be classified as "unknown primary" or "primary colonic melanomas" remains open.
